# Supplementary material for: Chromothripsis during telomere crisis is independent of NHEJ, and consistent with a replicative origin
Source: Genome Res. 2019 May;29(5):737–49. doi: 10.1101/gr.240705.118 (PMC6499312; doi:10.1101/gr.240705.118)
Supplement: Supplemental Material [file supp_gr.240705.118_Supplemental_file_1.zip › contigs/annotated_contigs/DB79/contig.2.DB79_length_751_mean_cov_10.585885486.docx]

**DB79_length_751_mean_cov_10.585885486**

```
ATGTTTCACCTTGATAAAATAGTCTTAGGGTAAGGAAAAAATAGAATTTCATAGAAGATAGTCTATTTTTTCTTTCCTGAAGAGTTAAC
 >chr10:133118365-133118750 + E=8e-220 p=1e-02
TTAAATAATTCTGTGCATTCAGGGAATTCTGCCTCTTAACCTTTCCTTTGACTCTTCCTGCTCATTAATAAATACATTGAGATATGCTT

GCCTTGGGACCAGTCCCTGGGGCATCTGACTTCTGACCTTTTTCCATGTTCAGAATTAGATGGGGCAAGAGTTTTGATTATCTAAGGAG

CTAAGAGGTAAAATAGAAATACGCCCATGAGACTGTGGGGCCTGAGAGAGAATAGGGTTTCACGGGGGTCTGGGAGGGCAGCGGGGCCA

CCTCAAGCTGAATCACCTGCTCTGTGGCT|CCGGCTTTATTTCACAAGCGGACACAAGTAAGCAACGGGCAAAACCTCCCCCGAGGGAA
                               >chr10:133036758-133037124 + E=2e-208
CGGCTCCTGGAAGTTATGTGCTTTCCTATTGGGAACCTTCCTCCATGTTCATTATCTCTGGTGCTTGTTCAAGCTTGCTGCGGTTCTTT

GTGAAATAGGAAGTCGCCCTTCCATGTGCAGCCCTGGCAAAGATTGTTCTGGAAGTGCTGATGGTCTCGGGCTGGAAATGGGACCTGGT

CCTCTTGCCAAATGGTCCAAGCCACTTGCCCATCACGCGGGCAGAGCTGAAGGGGAAGAAGAGGCTGGGCCGCAGCTTTGACTCGGCGC

TCAGGCATTTTCTCTGCCTGGCAGTGCTGGGAAAAGGTAC
```
